# Clinical and imaging outcome of osteochondral lesions of the talus treated using autologous matrix-induced chondrogenesis technique with a biomimetic scaffold

**DOI:** 10.1186/s12891-017-1679-x

**Published:** 2017-07-18

**Authors:** Domenico Albano, Nicolò Martinelli, Alberto Bianchi, Carmelo Messina, Francesco Malerba, Luca Maria Sconfienza

**Affiliations:** 1 Department of Radiology, Di.Bi.Med, University of Palermo, Via del Vespro 127, 90127 Palermo, Italy; 2 Department of Foot and Ankle Surgery, IRCCS Istituto Ortopedico Galeazzi, Via Riccardo Galeazzi 4, 20161 Milano, Italy; 3 Scuola di Specializzazione in Radiodiagnostica, Università degli Studi di Milano, Via Festa del Perdono 7, 20122 Milano, Italy; 4 Unit of Diagnostic and Interventional Radiology, IRCCS Istituto Ortopedico Galeazzi, Via Riccardo Galeazzi 4, 20161 Milano, Italy; 5 Department of Biomedical Sciences for Health, Università degli Studi di Milano, Via Festa del Perdono 7, 20122 Milano, Italy

**Keywords:** Osteochondral lesion, Talus, Scaffold, Cartilage, Magnetic resonance imaging

## Abstract

**Background:**

The purpose of our study was to assess the clinical and imaging outcome of autologous matrix-induced chondrogenesis (AMIC) technique consisting of microfractures followed by the filling of osteochondral lesions of the talus (OLTs) with a cell-free biphasic collagen-hydroxyapatite osteochondral scaffold (MaioRegen).

**Methods:**

Sixteen patients (eight males, age: 42.6 ± 18.4, range 14–74) with OLT repaired using AMIC technique, with implantation of MaioRegen, were clinically evaluated through the American Orthopedic Foot and Ankle Society Score (AOFAS) and a 10-point Visual Analogue Scale (VAS) pain score after a mean follow-up of 30 ± 16.9 months. The MRI examinations were performed 12 and 24 months after surgery. A paired t-test was applied to compare pre- and post-operative clinical findings (VAS and AOFAS) and Magnetic resonance observation of cartilage repair tissue (MOCART) score changes in the follow-up. To assess the correlation between variation of AOFAS and MOCART scores, the Pearson’s correlation coefficient was calculated.

**Results:**

No complications after surgery were encountered. From pre-operative to post-operative values, there was a significant (*P* < 0.001) reduction of mean VAS pain score (6.3 ± 0.9,range: 4–8 and 2.9 ± 1.8,range: 0–6, respectively) and increase of AOFAS score (60.2 ± 7.8,range: 50–74 and 77.4 ± 16.2,range: 50–100, respectively). Among 16 patients, six (37%) were not satisfied at the end of follow-up, six (37%) were moderately satisfied and four (25%) were highly satisfied. The treatment was considered failed in five out of 16 patients (31%). Among them, four (25%) required re-interventions with implantation of ankle prostheses, whereas one patient was treated with a further AMIC technique combined with autologous bone graft and platelet-rich plasma. The mean MOCART score was 41.9 ± 14.6 (25–70) 12 months after surgery and 51.9 ± 11.6 (30–70) after 24 months, with a statistically significant increase (*P* = 0.012). However, no correlation was seen between AOFAS and MOCART changes (*r* = 0.215, *p* = 0.609).

**Conclusion:**

The high rates of treatment failure encountered in our study using MaioRegen need to be confirmed by larger studies and should induce the scientific community questioning the reliability of this biomimetic scaffold for the treatment of OLTs.

## Background

Osteochondral lesions of the talus (OLT) are defects of the chondral layer and subchondral bone, which commonly affect the talar articular surface. Their etiology remains unclear, although a relation with post-traumatic instability of the ankle has been postulated [1]. OLT may determine a variable clinical picture, ranging from incidental diagnosis in asymptomatic patients to severe pain and limitation of daily activities [2]. When incidentally discovered, OLTs may be treated conservatively with rest, nonsteroidal anti-inflammatory drugs, and intra-articular injections of platelet-rich plasma or hyaluronic acid, although conservative therapies are usually ineffective thereby requiring surgery [3]. To date, several surgical approaches have been proposed to treat OLT, including osteochondral grafting, debridement, microfractures an chondrocyte implantation, even though there is no consensus on the best treatment choice [4].

Autologous matrix-induced chondrogenesis (AMIC) is a procedure consisting of microfractures followed by osteochondral defect filling using a scaffold that allows the regeneration of articular cartilage from bone marrow mesenchymal stem cells [5]. For reconstruction, tissue engineering should consider to support the regeneration of both cartilage and subchondral bone. To date, almost all of the scaffolds present in the market are homogeneous and cannot balance chondrogenesis and osteogenesis simultaneously for repairing osteochondral defects [6]. Therefore, new biphasic scaffolds with different layers mimicking the structures of osteochondral tissues have been designed to close this chasm. The MaioRegen is a cell-free biometic scaffold, which with its structure can address both the chondral layer and the underlying subchondral bone. Furthermore, MaioRegen is implanted with a one-step technique, which can theoretically decreases surgical time, costs and morbidity. Controversial results have been reported for osteochondral defect repair in the knee [7, 8].

After surgery, conventional radiographs and computed tomography allows evaluating only the bony component without providing any information regarding cartilage layer. Conversely, magnetic resonance imaging (MRI) is a radiation-free modality, which is considered the reference standard to assess the osteochondral lesions after surgical repair [9]. The Magnetic Resonance Observation of Cartilage Repair Tissue (MOCART) score is conventionally used to grade cartilage regeneration over time [10]. It was introduced to evaluate the repair tissue after treatment of osteochondral lesions of the knee [10–12]. Nevertheless, several authors have used this score in the ankle to monitor the healing of OLTs repaired with AMIC technique achieving controversial results in the correlation between clinical scores and MOCART values after surgery [13–17].

The purpose of our study was to assess the clinical and MRI outcome of AMIC technique performed with a cell-free biphasic collagen-hydroxyapatite osteochondral scaffold for the treatment of OLT. Moreover, we evaluated the correlation existing between clinical and MOCART scores to investigate the role of imaging in these patients.

## Methods

### Patients

This retrospective study was approved by Ospedale San Raffaele (Milano, Italy) Ethical Committee (Protocol #RM106). All individuals involved in this study provided written consent to use their clinical and imaging data for research purposes. This study has been conducted according to the principles expressed in the Declaration of Helsinki.

All OLTs were diagnosed using MRI after being suspected based on clinical findings. Inclusion criteria for the surgical procedure were symptomatic OLTs classified as type II or IIA (>1.5 cm^2^ in area and >5 mm deep, respectively) [18, 19], with history of failed conservative treatment. Exclusion criteria were: history of ankle fracture, hemophilia, pregnancy, septic ankle arthritis, rheumatic diseases and neuromuscular disorders.

### AMIC technique

All patients were treated by two orthopedic surgeons specialized in ankle surgery (N.M. and A.B.). Lesions were approached through a medial malleolar osteotomy. The line of osteotomy was performed at the junction of the medial plafond to obtain adequate exposure of the lesion. The site was prepared by creating a defect with stable shoulders where the scaffold was placed. Microfractures were performed using a chondral pick. The lesion was templated using an aluminum foil, and the scaffold (MaioRegen, FinCeramica Faenza, Faenza, Italy) was cut to the exact size of the defect and implanted by press-fitting. This osteochondral biomimetic scaffold has a porous three-dimensional composite three-layer structure that mimics the osteochondral anatomy [20]. After the scaffold was implanted, osteotomy was fixed with two malleolar screws that were inserted through predrilled holes. Patients were immobilised in a non-weight-bearing plaster cast for 4 weeks; the duration of casting was extended depending on radiological consolidation of the malleolar osteotomy. After removal of the cast the patients started light weight bearing with two crutches for 4 weeks.

### Clinical evaluation

The clinical evaluation was performed through the American Orthopedic Foot and Ankle Society Score (AOFAS) (poor <70 points, fair 70–79 points, good 80–89 points, excellent 90–100 points) [21]. The AOFAS score is a clinician-administered questionnaire, which includes three areas: pain, function and alignment. Although this score was developed specifically to assess foot or ankle problems, some aspects of quality of life can be evaluated with this outcome measure. A 10-point Visual Analogue Scale (VAS) for the assessment of patient’s pain was also performed. Clinical evaluation was made up to 15 days before surgery and after treatment. The validated Italian version of Foot Function Index (FFI) [22] was also applied but only for post-operative evaluation, as this score was validated after that most of our patients were surgically treated. The FFI consists of 18 items grouped into two subscales: pain (FFI-P) and disability (FFI-D). Each of the two subscales is calculated as the sum of the items included. Scores are then transformed to a 0–100 scale. The measure generates two separate scores (FFI-P, FFI-D) where the higher the score, the worst the health state.

At the end of follow-up, each patient was asked to express his overall satisfaction as: (i) not satisfied; (ii) moderately satisfied; (iii) highly satisfied. The surgical intervention was considered failed in case of persistent pain with VAS > 4 and the patients needed to be re-operated for residual pain.

### MRI protocol and image interpretation

All MRI scans of the ankle were performed on one of two 1.5 T MRI scanners (Avanto and Espree, Siemens Medical Solution, Erlangen, Germany) using a dedicated extremity coil. The MRI examinations were performed 12 and 24 months after surgery in a subset of patients (*n* = 8). In our MRI protocol the following sequences were included: sagittal T1-weighted turbo spin-echo, sagittal short time inversion recovery, axial T1-weighted turbo spin-echo, axial proton density-weighted fat-saturated, coronal proton density-weighted fat-saturated, coronal T2-weighted turbo spin-echo (slice thickness 3 mm).

One musculoskeletal radiologist with 12 years’ experience reviewed the MRI scans of the ankle performed after treatment to evaluate the MOCART score. In our institution, radiologists and orthopaedic surgeons, are trained in the use of MOCART score since the cases of OLTs surgically repaired are frequently discussed in multidisciplinary sessions. This score is composed of nine variables, which are used to describe cartilage repair tissues after treatment: (i) defect filling, (ii) cartilage interface, (iii) surface, (iv) adhesions, (v) structure, (vi) signal intensity, (vii) subchondral lamina, (viii) subchondral bone, (ix) effusion [10]. A score is given for all variables and the total score ranges from 0 (worse condition) to 100 points (best condition).

### Statistical analysis

Nomality of data distribution was verified. A paired t-test was applied to compare pre- and post-operative clinical findings and MOCART changes in the follow-up. The t-test was used after checking normal distribution of data. To assess the correlation between variation of AOFAS and MOCART scores, the differences between pre- and post-treatment values (change in AOFAS and change in MOCART) were calculated. Then, the Pearson’s correlation coefficient was calculated to better understand the relationship between clinical findings and MRI features and the role of imaging in monitoring these patients. Statistical analysis was performed using SPSS® software (v. 23, IBM, Armonk, New York, NY). A *P*-value lower than 0.05 was considered as statistically significant, where appropriate.

## Results

Among 62 patients with OLT, 16 were treated with the AMIC technique using the biomimetic osteochondral scaffold MaioRegen and met the inclusion criteria, thereby being enrolled in this study. Thus, our study population included 16 patients (8 males, 8 females; age: 42.6 ± 18.4, range 14–74) with OLT repaired using the AMIC technique with MaioRegen implantation between January 2013 and June 2016. The patients had a mean body mass index of 26.3 ± 5.2 (range: 19.1–40.5).

No complications after surgery were encountered. Mean follow-up of our patients was 30 ± 16.9 months (range: 10–52).

Pain decrease in terms of VAS was observed in 15/16 patients (94%) while it remained unchanged in one patient (6%). There was a significant (*P* < 0.001) reduction of mean VAS pain score from pre-operative values (6.3 ± 0.9, range: 4–8) to post-operative values (2.9 ± 1.8, range: 0–6).

Pre-operative AOFAS score was poor in 15/16 patients (94%) whereas it was fair in one patient (6%). After surgery, the AOFAS score was poor in six patients (37%), fair in two patients (12%), and excellent in the remaining eight patients (50%). We found a statistically significant increase (*P* < 0.001) between pre-operative (60.2 ± 7.8, range: 50–74) and post-operative (77.4 ± 16.2, range: 50–100) AOFAS scores.

Compared to baseline, at the end of follow-up the AOFAS scores improved in 11/16 patients (69%), remained unchanged in 4/16 patients (25%) and worsened in one patient (6%). The mean post-operative FFI-D and FFI-*P* values were 38.2 ± 29.1 and 31.1 ± 27.1, respectively.

Among 16 patients, six (37%) were not satisfied at the end of follow-up, six (37%) were moderately satisfied, and four (25%) were highly satisfied.

The treatment was considered failed in five out of 16 patients (31%). Among them, four (25%) required re-interventions with implantation of ankle prostheses (HemiCAP®), whereas one patient was treated with a further AMIC technique combined with autologous bone graft and platelet-rich plasma.

**Fig. 1:**
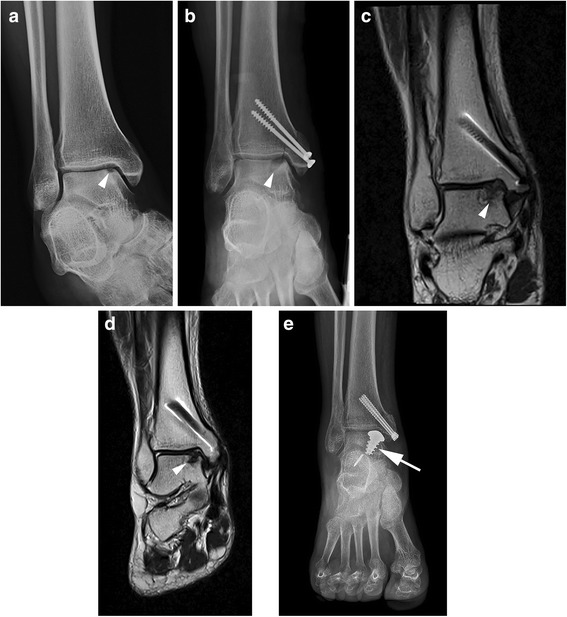
Fifty-three year old woman with OLT repaired using AMIC technique with MaioRegen. Preoperative (**a**) and postoperative (**b**) conventional radiographs show a medial talar dome osteochondral lesion (*headarrows*). Coronal T2-weighted turbo spin-echo images at 12 months (**c**) and 24 months (**d**) show complete filling of the osteochondral defect of the medial talar dome (*headarrows*). Conventional radiograph performed after re-intervention (**e**) with implantation of talar dome HemiCAP® (*arrow*)

The mean MOCART score was 41.9 ± 14.6 (25–70) 12 months after surgery and 51.9 ± 11.6 (30–70) after 24 months, with an improvement of 24% and a statistically significant increase (*P* = 0.012). However, no correlation was seen between change in AOFAS and change in MOCART (*r* = 0.215, *p* = 0.609). The MOCART score improved in 5/8 patients (63%), whereas it remained unchanged in 3 patients, and complete filling of the osteochondral defect at the level of the surrounding cartilage was observed in 3/8 patients (37%). Figure 1 shows a representative case from our series.

## Discussion

Our main finding was that the AMIC technique with MaioRegen implant failed in 31% of our patients who required re-intervention with implantation of ankle prostheses, although we found a progressive significant improvement of AOFAS, VAS and MOCART scores overall.

It is not easy to compare our results to those of previous studies focusing on other surgical treatments of OLTs, since different criteria for considering successful outcomes can be encountered. Chuckpaiwong et al. studied 105 patients with OLT treated with arthroscopic debridement with osteochondral bone stimulation (microfracture) [23]. They observed a successful rate of 70% with all unsuccessful treatments involving patients with lesions greater than 15 mm [23]. Ferkel and colleagues evaluated the long-term (average follow-up: 71 months) results of patients with chronic symptomatic OLT treated predominantly by arthroscopic excision and drilling ankle. They reported good to excellent results in 64% of patients based on the modified Weber score, and 72% of the patients with the Alexander score. The average AOFAS score was 84 in their series in line with other studies on arthroscopic treatment of OLT [24]. Autologous chondrocyte implantation has shown to be an effective procedure for the treatment of OLT with excellent or good clinical results in 90% of patients on a 10-year follow-up study by Giannini et al. [25]. Regarding the arthroscopic treatment with bone marrow-derived mesenchymal stem cell transplantation, Giannini and colleagues demonstrated the good clinical results at 48 months’ follow-up with 78% of patients being able to resume previous sports level [17].

Over the last years, several studies have focused on the treatment of osteochondral lesions with procedures that induce the restoration of articular surface using tissue-engineering technology and scaffold implantation [7, 20, 26–31]. MaioRegen is a multilayered biomimetic scaffold consisting of collagen type I and magnesium-hydroxyapatite, which should induce cartilage and bone regeneration [26]. In previous studies, mainly evaluating the repair of osteochondral knee defects, the use of MaioRegen has shown to lead to promising clinical results [7, 20, 30, 31]. Nevertheless, Christensen et al. have arisen concerns about the use of this scaffold for the treatment of osteochondral lesions, suggesting using it with caution [8]. Although they found a clinical improvement in patients with osteochondral lesions of femoral condyle, patella and talus, they underlined the poor results achieved in terms of subchondral bone and cartilage restoration. As well as with MRI, they monitored patients with computed tomography that allowed them to better demonstrate the insufficient bone formation within the osteochondral defect [8]. Similarly to them, we found an improvement of clinical findings after surgery with good post-operative FFI values and a significant increase of AOFAS and decrease of VAS scores. However, we encountered poor patients’ satisfaction and high rates of treatment failure. Thus, we support the hypothesis of Christensen and colleagues that the initial clinical improvement of patients with osteochondral lesions treated with MaioRegen could be related to the removal of the affected subchondral bone rather than cartilage repair or bone restoration, since in osteochondral lesions pain arises from subchondral bone [8]. The incomplete recovery of both cartilage layer and subchondral bone after surgery could probably lead to a subsequent worsening of these structures status.

In a previous study, Valderrabano et al. reported an adapted AMIC technique to treat OLTs, which included the addiction of mesenchymal stem cells-rich autologous cancellous bone from the iliac crest to improve the reconstruction of bony defect [10]. Twenty-four months after surgery, they obtained a significant improvement in AOFAS and VAS scores. They also found a complete filling of the osteochondral defect at the level of the surrounding cartilage in MRI scans performed in 35% of patients. We saw a complete filling of the defect in 37% of our patients, in line with the results of the study of Valderrabano [13], even without the use of any mesenchymal cell supplementation.

Kubosch et al. demonstrated a significant improvement in post-operative clinical and imaging findings in 17 patients with OLTs treated with autologous subchondral cancellous bone graft and AMIC [14]. After a mean follow up of 39.5 ± 18.4 months, they also found a significant correlation between MOCART and AOFAS score [14]. In our study, MOCART score showed improvement of imaging findings after surgery. However, the increase of MOCART score was not correlated with AOFAS score changes. Similarly to our results, Aurich et al. did not find any correlation between MOCART and clinical scores after treatment with AMIC technique for OLTs [16] whereas, as we reported above, Kubosch et al. found a significant correlation [14]. As suggested by Christensen and colleagues, a thorough radiological follow-up for patients with OLTs repaired using biomimetic scaffolds is recommended [8]. Nevertheless, although MRI can be considered useful to identify eventual complications after surgical repair of OLTs, the assessment of the cartilage status with standard sequences may be challenging. The MOCART score was specifically designed to evaluate chondral repair of the knee, where cartilage thickness and joint space is larger. In the ankle, the cartilage layer and the joint space is smaller compared to the knee, thus making the use of MOCART suboptimal in this setting. Thus, other available options, which deserve to be considered in future studies on the biological cartilage repair, are the delayed Gadolinium-enhanced MRI of cartilage [32] and MRI-specific sequences [12, 33, 34]. Among them, the most promising are T1 rho imaging, T2 mapping, diffusion-weighted imaging and diffusion tensor imaging, which might help to better evaluate the cartilage ultrastructure after surgery [12, 33, 34].

Some limitations should be taken into account. First, this is a retrospective study with relatively small number of patients. Second, the MRI protocol did not include three-dimensional fat-suppressed gradient echo acquisition that may have improved the reliability of the MOCART score. However, a similar protocol was also used in previous papers on the topic [35]. Then, we have not investigated the reproducibility of the MOCART score. However, recently MOCART score has not shown to be reproducible for the evaluation of surgical repaired OLTs [36]. Last, another limitation of the work might be the absence of control group, although our aim was mainly to evaluate clinical efficacy and imaging outcome over time.

## Conclusions

In conclusion, we found a high rate of failures in patients treated using MaioRegen for OLTs. However, this data needs to be confirmed by larger studies to better understand the applicability of this biomimetic scaffold for the treatment of OLTs. Although a thorough radiological follow-up is recommended in patients with surgically-treated OLTs, the MOCART score does not correlate with the clinical data.

## Abbreviations

AMIC: Autologous matrix-induced chondrogenesis
FFI: Foot Function Index
MOCART: Magnetic resonance observation of cartilage repair tissue
MRI: Magnetic resonance imaging
OLT: Osteochondral lesions of the talus
VAS: Visual analogue scale

## References

[1] Uozumi H, Sugita T, Aizawa T, Takahashi A, Ohnuma M, Itoi E (2009). Histologic findings and possible causes of osteochondritis dissecans of the knee. Am J Sports Med.

[2] van Dijk CN, Reilingh ML, Zengerink M, van Bergen CJ (2010). Osteochondral defects in the ankle: why painful?. Knee Surg Sports Traumatol Arthrosc.

[3] Vannini F, Costa GG, Caravelli S, Pagliazzi G, Mosca M (2016). Treatment of osteochondral lesions of the talus in athletes: what is the evidence?. Joints..

[4] Oussedik S, Tsitskaris K, Parker D (2015). Treatment of articular cartilage lesions of the knee by microfracture or autologous chondrocyte implantation: a systematic review. Arthroscopy.

[5] Lee YH, Suzer F, Thermann H (2014). Autologous matrix-induced chondrogenesis in the knee: a review. Cartilage.

[6] Li X, Ding J, Wang J, Zhuang X, Chen X (2015). Biomimetic biphasic scaffolds for osteochondral defect repair. Regen Biomater.

[7] Perdisa F, Filardo G, Sessa A, Busacca M, Zaffagnini S, Marcacci M (2017). One-step treatment for patellar cartilage defects with a cell-free Osteochondral scaffold. Am J Sports Med.

[8] Christensen BB, Foldager CB, Jensen J, Jensen NC, Lind M (2016). Poor osteochondral repair by a biomimetic collagen scaffold: 1- to 3-year clinical and radiological follow-up. Knee Surg Sports Traumatol Arthrosc.

[9] Recht M, White LM, Winalski CS, Miniaci A, Minas T, Parker RD (2003). MR imaging of cartilage repair procedures. Skelet Radiol.

[10] Marlovits S, Striessnig G, Resinger CT, Aldrian SM, Vecsei V, Imhof H (2004). Definition of pertinent parameters for the evaluation of articular cartilage repair tissue with high-resolution magnetic resonance imaging. Eur J Radiol.

[11] Sofu H, Kockara N, Oner A, Camurcu Y, Issin A, Sahin V (2017). Results of Hyaluronic Acide-based cell-free scaffold application in combination with microfracture for the treatment of Osteochondral lesions of the knee: 2-year comparative study. Arthroscopy.

[12] Trattnig S, Ohel K, Mlynarik V, Juras V, Zbyn S, Korner A (2015). Morphological and compositional monitoring of a new cell-free cartilage repair hydrogel technology-GelrinC by MR using semi-quantitative MOCART scoring and quantitative T2 index and new zonal T2 index calculation. Osteoarthr Cartil.

[13] Valderrabano V, Miska M, Leumann A, Wiewiorski M (2013). Reconstruction of osteochondral lesions of the talus with autologous spongiosa grafts and autologous matrix-induced chondrogenesis. Am J Sports Med.

[14] Kubosch EJ, Erdle B, Izadpanah K, Kubosch D, Uhl M, Südkamp NP (2016). Clinical outcome and T2 assessment following autologous matrix-induced chondrogenesis in osteochondral lesions of the talus. Int Orthop.

[15] Lee KT, Choi YS, Lee YK, Cha SD, Koo HM (2011). Comparison of MRI and arthroscopy in modified MOCART scoring system after autologous chondrocyte implantation for osteochondral lesion of the talus. Orthopedics.

[16] Aurich M, Bedi HS, Smith PJ, Rolauffs B, Mückley T, Clayton J (2011). Arthroscopic treatment of osteochondral lesions of the ankle with matrix-associated chondrocyte implantation: early clinical and magnetic resonance imaging results. Am J Sports Med.

[17] Giannini S, Buda R, Battaglia M, Cavallo M, Ruffilli A, Ramponi L (2013). One-step repair in talar osteochondral lesions: 4-year clinical results and t2-mapping capability in outcome prediction. Am J Sports Med.

[18] Giannini S, Buda R, Cavallo M, Ruffilli A, Cenacchi A, Cavallo C (2010). Cartilage repair evolution in post-traumatic osteochondral lesions of the talus: from open field autologous chondrocyte to bone-marrow-derived cells transplantation. Injury.

[19] Giannini S, Buda R, Faldini C, Vannini F, Bevoni R, Grandi G (2005). Surgical treatment of osteochondral lesions of the talus in young active patients. J Bone Joint Surg Am.

[20] Kon E, Delcogliano M, Filardo G, Pressato D, Busacca M, Grigolo B (2010). A novel nano-composite multi-layered biomaterial for treatment of osteochondral lesions: technique note and an early stability pilot clinical trial. Injury.

[21] Kitaoka HB, Alexander IJ, Adelaar RS, Nunley JA, Myerson MS, Sanders M (1994). Clinical rating systems for the ankle-hindfoot, midfoot, hallux, and lesser toes. Foot Ankle Int.

[22] Martinelli N, Scotto GM, Sartorelli E, Bonifacini C, Bianchi A, Malerba F (2014). Reliability, validity and responsiveness of the Italian version of the foot function index in patients with foot and ankle diseases. Qual Life Res.

[23] Chuckpaiwong B, Berkson EM, Theodore GH (2008). Microfracture for Osteochondral lesions of the ankle: outcome analysis and outcome predictors of 105 cases. Arthroscopy.

[24] Ferkel RD, Zanotti RM, Komenda GA, Sgaglione NA, Cheng MS, Applegate GR (2008). Arthroscopic treatment of chronic osteochondral lesions of the talus: long-term results. Am J Sports Med.

[25] Giannini S, Battaglia M, Buda R, Cavallo M, Ruffilli A, Vannini F (2009). Surgical treatment of osteochondral lesions of the talus by open-field autologous chondrocyte implantation: a 10-year follow-up clinical and magnetic resonance imaging T2-mapping evaluation. Am J Sports Med.

[26] Kon E, Delcogliano M, Filardo G, Fini M, Giavaresi G, Francioli S (2009). Orderly osteochondral regeneration in a sheep model using a novel nano-composite multilayered biomaterial. J Orthop Res.

[27] Filardo G, Kon E, Berruto M, Di Martino A, Patella S, Marcheggiani Muccioli GM (2012). Arthroscopic second generation autologous chondrocytes implantation associated with bone grafting for the treatment of knee osteochondritis dissecans: results at 6 years. Knee.

[28] Filardo G, Kon E, Andriolo A, Di Martino A, Zaffagnini S, Marcacci M (2014). Treatment of “Patellofemoral” cartilage lesions with matrix-assisted Autologous Chondrocyte transplantation: a comparison of patellar and Trochlear lesions. Am J Sports Med.

[29] Filardo G, Andriolo L, Sessa A, Vannini F, Ferruzzi A, Marcacci M (2017). Age is not a contraindication for cartilage surgery: a critical analysis of standardized outcomes at long-term follow-up. Am J Sports Med.

[30] Filardo G, Kon E, Perdisa F, Di Matteo B, Di Martino A, Iacono F (2013). Osteochondral scaffold reconstruction for complex knee lesions: a comparative evaluation. Knee.

[31] Kon E, Delcogliano M, Filardo G, Altadonna G, Marcacci M (2009). Novel nano-composite multi-layered biomaterial for the treatment of multifocal degenerative cartilage lesions. Knee Surg Sports Traumatol Arthrosc.

[32] Samosky J, Burstein D, Eric Grimson W, Howe R, Martin S, Gray ML (2005). Spatially-localized correlation of dGEMRIC-measured GAG distribution and mechanical stiffness in the human tibial plateau. J Orthop Res.

[33] Potter HG, Black BR, Chong LR (2009). New techniques in Articular cartilage imaging. Clin Sports Med.

[34] Ukai T, Sato M, Yamashita T, Imai Y, Mitani G, Takagaki T (2015). Diffusion tensor imaging can detect the early stages of cartilage damage: a comparison study. BMC Musculoskelet Disord.

[35] Usuelli FG, Grassi M, Manzi L, Guarrella V, Boga M, De Girolamo L (2016). Treatment of osteochondral lesions of the talus with autologous collagen-induced chondrogenesis: clinical and magnetic resonance evaluation at one-year follow-up. Joints.

[36] Albano D, Martinelli N, Bianchi A, Giacalone A, Sconfienza LM. Evaluation of reproducibility of the MOCART score in patients with osteochondral lesions of the talus repaired using the autologous matrix-induced chondrogenesis technique. Radiol Med. 2017. In press. 10.1007/s11547-017-0794-y 28770483

